# ^1^H-NMR-Based Metabonomics Study to Reveal the Progressive Metabolism Regulation of SAP Deficiency on ApoE^−/−^ Mice

**DOI:** 10.3390/metabo12121278

**Published:** 2022-12-16

**Authors:** Qian Li, Wanting Chen, Wenbin Huang, Ranran Hou, Xinping Huang, Man Xu, Limei Que, Lijing Wang, Yongxia Yang

**Affiliations:** 1 School of Life Sciences and Biopharmaceutics, Guangdong Pharmaceutical University, Guangzhou 510006, China; 2 School of Pharmacy, Guangdong Pharmaceutical University, Guangzhou 510006, China; 3 Department of Breast Care Surgery, The First Affiliated Hospital, Guangdong Pharmaceutical University, Guangzhou 510006, China; 4 Foshan Fosun Chancheng Hospital, Foshan 528031, China; 5 School of Medical Information and Engineering, Guangdong Pharmaceutical University, Guangzhou 510006, China; 6 Guangdong Province Key Laboratory for Biotechnology Drug Candidates, Guangzhou 510006, China

**Keywords:** atherosclerosis, SAP, ^1^H-NMR, metabonomics

## Abstract

Atherosclerosis is the most common disease of the vascular system and the metabolic disorder is one of its important molecular mechanisms. SAP protein is found to be highly expressed in atherosclerotic blood vessels. Our previous study found that SAP deficiency can significantly inhibit the development of atherosclerosis. However, the regulatory effect of SAP deficiency on AS metabolism is unknown. Based on ^1^H-NMR metabonomics, this study investigated the serum metabolic changes in ApoE^−/−^;SAP^−/−^ mice compared with ApoE^−/−^ mice during the whole progression of atherosclerosis. The results showed that acetate, pyruvate, choline and VLDL + LDL were statistically regulated to the normal levels as in C57 mice by SAP deficiency in ApoE^−/−^;SAP^−/−^ mice at 8 w (without obvious plaques). With the appearance and aggravation of atherosclerotic plaques (8 + 4 w and 8 + 8 w), the four metabolites of acetate, pyruvate, choline and VLDL + LDL were continuously regulated, which were denoted as the metabolic regulatory markers of SAP deficiency. We also found that the changes in these four metabolites had nothing to do with high-fat diet. Therefore, it was revealed that SAP deficiency regulated the metabolic disorders in ApoE^−/−^ prior to the appearance of obvious atherosclerotic plaques, which is one of the important mechanisms leading to the inhibition of atherosclerosis, providing a new basis for the application of SAP in atherosclerosis.

## 1. Introduction

Atherosclerosis is one of the most common diseases in the vascular system, and it can further lead to coronary heart disease, cerebral thrombosis, infarction and other related diseases. As the pathological basis of these diseases, atherosclerosis has seriously threatened people’s health [1].

Serum amyloid P protein (SAP), as an acute phase response protein, plays a crucial role in the body’s innate immune and inflammatory responses [2]. Clinical studies have found that SAP is significantly positively correlated with the pathological process of atherosclerosis [3]. SAP does not exist in the normal vascular intima, but it is specifically located in the atherosclerotic intima [4]. Relevant animal experiments have shown that SAP binds with high-density lipoprotein (HDL) and very low-density lipoprotein (VLDL) with high affinity, indicating that SAP is involved in atherosclerosis and lipoprotein metabolism [5]. In-situ hybridization and RT-PCR analysis indicated that SAP is partially produced by macrophages and smooth muscle cells in neointima in atherosclerotic lesions [6]. Recent study has further demonstrated that SAP may participate in cholesterol clearance by promoting cholesterol efflux [7]. Our group found that deletion of the SAP gene can significantly inhibit the formation of atherosclerosis and the aggregation of foam cells in ApoE^−/−^ mice. We further examined the expression of several macrophage transporters and the result suggested that SAP may be involved in SR-BI-mediated cholesterol efflux [8].

The occurrence of atherosclerotic disease can cause disorders of multiple metabolic pathways such as glucose metabolism and lipid metabolism [9,10]. Clinical studies have found that in atherosclerotic plaque, tryptophan, purine, uric acid, glutathione (GSH), sphingolipids, and glycerophospholipids are involved in the metabolic imbalance of atherosclerosis [11]. It was found that the metabolism of bile acids and sterols is positively correlated with the pathological process of atherosclerosis in a rabbit model of atherosclerosis [12]. Using GC-MS and ^1^H-NMR, Joanna Teul et al. found that citric acid, succinic acid and malic acid in the TCA cycle showed a downward trend in the serum of patients with clinical atherosclerosis [13]. Christelle Guillermier et al. used multi-isotope imaging mass spectrometry (MIMS) to show significant heterogeneity of glucose metabolism in vascular smooth muscle cells in atherosclerosis [14]. The previous study demonstrated the inhibition of SAP knockout on atherosclerosis [8], but how the SAP gene affects the metabolic changes in atherosclerosis has not been reported.

Metabonomics is a comprehensive characterization of small molecule metabolites in cells, tissues, organs, and organisms, which can respond to metabolic changes caused by intrinsic or extrinsic factors. In recent years, metabonomics has developed rapidly, and it has become an important means to study related metabolic diseases [15]. ^1^H-NMR has become an effective technology in metabonomics research because it does not need to make special sample treatments and it is easy to operate. A study by Loanna Tzoulaki et al. found that atherosclerosis was significantly correlated with lipid metabolism, fatty acids, carbohydrates, aromatic amino acids and the TCA cycle by using ^1^H-NMR-based metabonomics [16]. The profiles of plasma NMR spectra of patients with coronary heart disease have been proven to have a predictive power of more than 90% for coronary heart disease [17]. Based on the method of ^1^H-NMR, we demonstrated that defects in the p-selectin glycoprotein ligand-1 (PSGL-1) gene can regulate glucose metabolism, lipid metabolism, amino acid and phospholipid metabolism in atherosclerosis at the metabolic level [18].

In this report, we performed ^1^H-NMR based metabonomics research to explore the metabolic changes in ApoE^−/−^;SAP^−/−^ mice during atherosclerosis progression and clarify the metabolic regulation of SAP deletion in atherosclerosis inhibition.

## 2. Materials and Methods

### 2.1. Laboratory Animals

The mice for the spontaneous atherosclerosis mouse model of ApoE^−/−^ were provided by Jackson Laboratory USA, serum amyloid P component deficient mice (SAP^−/−^) were provided by the Institute of Biochemistry and Cell Biology, and both groups of mice were on a C57 BL/6 background. ApoE^−/−^;SAP^−/−^ were generated by crossing ApoE^−/−^ mice with SAP^−/−^ mice, and double-gene-deficient mice (ApoE^−/−^;SAP^−/−^) in F1 progeny were identified by polymerase chain reaction (PCR) techniques. C57 mice were purchased from Guangdong Medical Laboratory Animal Center (GDMLAC). All mice were housed in an SPF-grade environment. There were 10 mice in each of the four groups. The common feed and high-fat feed (ordinary feed 87.5%, cholesterol 2%, bile salt 0.5%, lard 10%) consumed by animals were provided by GDMLAC. From 8 weeks, the normal feed was replaced with high-fat feed, and the consumption of high-fat feed was regularly checked. Feeding was stopped when the mice were 16 weeks old.

### 2.2. Blood Collection

Blood of mice at 8 w, 8 + 4 w (normal diet for 8 weeks and high-fat diet for 4 weeks) and 8 + 8 w (normal diet for 8 weeks and high-fat diet for 8 weeks) was drawn for use through the orbital venous plexus. After the mice were anesthetized routinely, a 1 cm long capillary tube was inserted under the eyeball of each mouse, and then the capillary tube was gently rotated until the blood flowed out of the capillary tube. Each mouse collected about 600 µL of blood, which was then stood for 2 h. Then, blood samples were centrifuged under 5000 rpm for 30 min to obtain serum. The serum samples were stored in the refrigerator at −80 °C.

### 2.3. ^1^H-NMR Spectroscopy

Serum samples were thawed and centrifuged at 4 °C for 10 min (12,000 rpm/min). A total of 250 µL of serum was added to a 5 mm NMR tube with 150 µL of PBS (0.2 mol/L, pH 7.4) and 100 µL D2O. Using a Bruker AVANCE III 500 MHz superconducting NMR spectrometer (Bruker, Inc., Billerica, MA, USA), serum ^1^H-NMR spectrum was acquired with a Carr–Purcell–Meiboom–Gill [CPMG, recycle delay-90-(τ-180-τ)_n_ –acquisition] pulse sequence. The probe temperature was 298 K. The total echo time was 100 ms (2nτ), the relaxation delay time was set to 3 s. The spectral width was 10 kHz, and the number of acquisitions was 128. Topspin 4.0 software was used for manual phase adjustment and baseline correction. Chemical shifts were calibrated with lactate doublet δ1.33. AMIX (V4.0.2, Bruker Biospin, Karlsruhe, Germany) was used to integrate peaks at δ 0.5–9.0. We set the integral at δ 4.7–5.5 to zero to remove the effect of the residual water signal, taking 0.04 ppm as the integration interval to carry out equal interval integration. The integrals were normalized to the sum of spectrum integrals and calculated the percentage of each peak area in the total peak area.

### 2.4. Pattern Recognition and Statistical Analysis

Simca-P+14.0 was used for pattern recognition analysis of integral data. All ^1^H-NMR spectral data were first observed in unsupervised mode using principal component analysis (PCA) and then further screened for metabolic differences between groups using orthogonal one-set partial least squares discriminant analysis (OPLS-DA). Variable importance in the projection (VIP) values greater than one were considered differential metabolic components. All metabolites were tested for significance using one-way ANOVA in SPSS 22.0 software.

## 3. Results

### 3.1. Metabolite Profiling of Serum Samples

**Figure 1:**
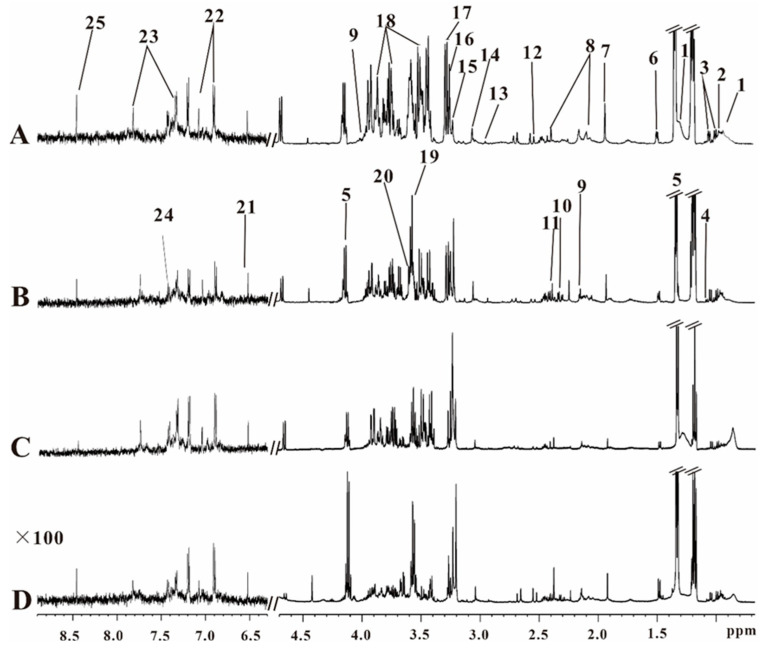
Representative serum ^1^H-NMR spectra of (**A**) ApoE^−/−^ mice, (**B**) ApoE^−/−^;SAP^−/−^ mice, (**C**) SAP^−/−^ mice, (**D**) C57 mice. (The spectral peaks in the range of δ 6.3–8.9 are amplified 100 times.) 1. VLDL + LDL. 2. leucine/isoleucine (Leu). 3. valine (Val). 4. α-ketoglutarate. 5. lactate (Lac). 6. alanine. 7. Acetate (Ace). 8. glutamine. 9. acetoacetate. 10. pyruvate(Pyr). 11. succinate(Suc). 12. citrate. 13. dimethylamine. 14. Creatine (Cr). 15. Choline (Cho). 16. TMAO. 17. Taurine (Tau). 18. α,β-glucose (α,β-Glc). 19. glycine(Gly). 20. glyceride. 21. fumarate. 22. Tyrosine (Try). 23. Histidine (His). 24. Phenylalanine (Phe). 25. formate.

Figure 1 showed the characteristic serum ^1^H-NMR spectra of the four different groups, i.e., ApoE^−/−^ mice (Figure 1A), ApoE^−/−^; SAP^−/−^ mice (Figure 1B), SAP^−/−^ mice (Figure 1C) and C57 mice (Figure 1D). Metabolites were assigned according to the references [19,20,21], the database of Biological Magnetic Resonance Data Bank (BMRB, https://bmrb.io/, on 1 October 2022) and the Human Metabolome Database (HMDB, https://hmdb.ca/, on 1 October 2022). We identified 25 metabolites based on the peak pattern of hydrogen spectrum and chemical shift. 

### 3.2. Multivariate Statistical Analysis

**Figure 2:**
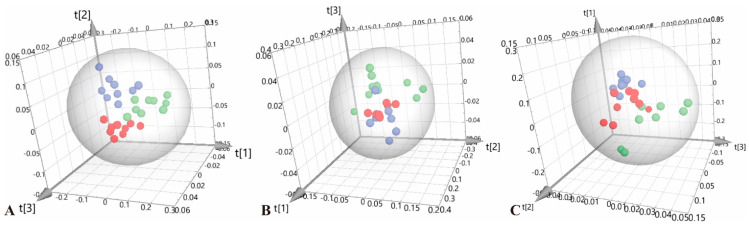
PCA scores plots for ^1^H-NMR spectra data. (**A**) 8 w, R^2^X = 0.937, Q^2^ = 0.904; (**B**) 8 + 4 w, R^2^X = 0.940, Q^2^ = 0.802; (**C**) 8 + 8 w, R^2^X = 0.955, Q^2^ = 0.778. ● C57; ● ApoE^−/−^; ● ApoE^−/−^;SAP^−/−^.

After normalizing, we performed principal component analysis (PCA) on the serum ^1^H-NMR spectra data of C57, ApoE^−/−^ and ApoE^−/−^;SAP^−/−^ mice at different experimental stages. The unsupervised discriminative mode PCA can observe the natural clustering trends among different groups, and the results were shown in Figure 2. The three groups were significantly separated at 8 w, 8 + 4 w and 8 + 8 w, indicating that the serum metabolic profiles were different among the three groups. We found that ApoE^−/−^;SAP^−/−^ mice were obviously separated from ApoE^−/−^ mice and tended to approach C57 mice, which showed that SAP deficiency can improve the metabolic disorders caused by atherosclerosis, to a certain extent.

**Figure 3:**
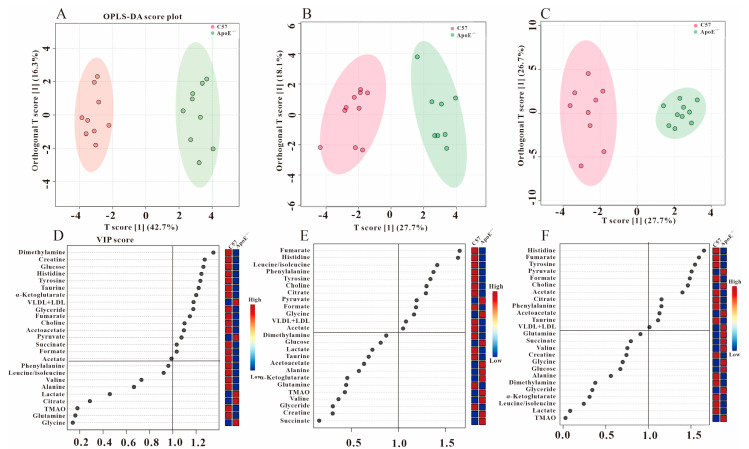
Metabolic differences between C57 and ApoE^−/−^ groups at three periods. OPLS−DA scores plots (**A**) (8 w), (**B**) (8 + 4 w), (**C**) (8 + 8 w) and VIP scores (**D**) (8 w), (**E**) (8 + 4 w), (**F**) (8 + 8 w).

**Figure 4:**
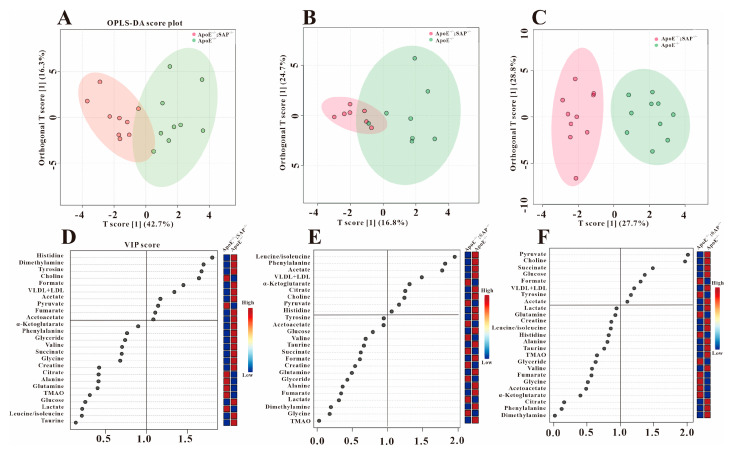
Metabolic differences between ApoE^−/−^;SAP^−/−^ and ApoE^−/−^ groups at three periods. OPLS−DA scores plots (**A**) (8 w), (**B**) (8 + 4 w), (**C**) (8 + 8 w) and VIP scores (**D**) (8 w), (**E**) (8 + 4 w), (**F**) (8 + 8 w).

**Figure 5:**
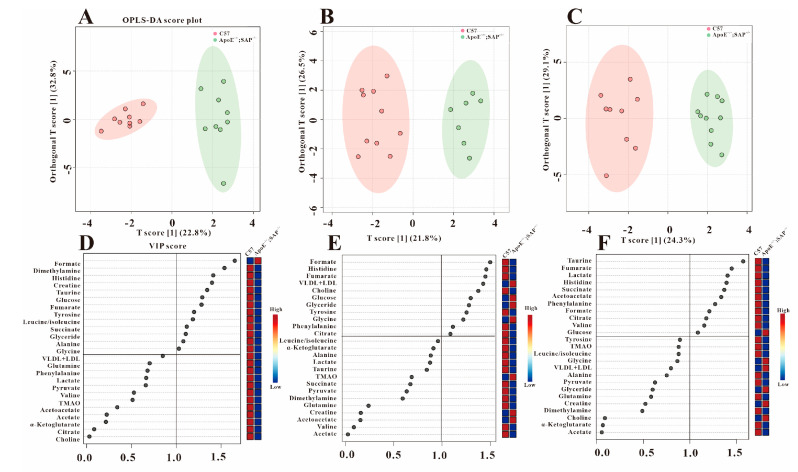
Metabolic differences between C57 and ApoE^−/−^;SAP^−/−^ groups at three periods. OPLS−DA scores plots (**A**) (8 w), (**B**) (8 + 4 w), (**C**) (8 + 8 w) and VIP scores (**D**) (8 w), (**E**) (8 + 4 w), (**F**) (8 + 8 w).

As a supervised discriminant analysis statistical method, orthogonal partial minimum discriminant analysis (OPLS-DA) was used to screen the metabolites’ differences. We performed OPLS-DA on the three groups at different periods (Figure 3, Figure 4 and Figure 5), and obtained the corresponding VIP values. The VIP value is the variable weight value of the OPLS-DA model, which can be used to measure the impact strength of the cumulative difference of each metabolite on the classification and discrimination of each group of samples. The VIP value greater than 1 is a common screening standard for differential metabolites. We used SPSS 22.0 to perform statistical analysis (*t*-tests) on the metabolites, and screened the differential metabolites by *p* value (*p* < 0.05) and the VIP value (VIP > 1).

### 3.3. Analysis of Serum Differential Metabolites in ApoE^−/−^ Mice

Our previous research proved that there were no obvious atherosclerotic plaques in the blood vessels of ApoE^−/−^ mice at 8 w [22], but the OPLS-DA scatter plot in Figure 3A shows the distinctive grouping between C57 and ApoE^−/−^ mice, indicating that the metabolic disorders have occurred in this stage without obvious atherosclerotic plaque. By statistical analysis of the metabolites with VIP > 1 (Figure 3D), we found that the seven metabolites of formate, tyrosine, fumarate, histidine, acetoacetate, acetate and choline were down-regulated, while pyruvate and VLDL + LDL increased statistically (Table 1).

Significant atherosclerotic plaque appeared in the coronary artery and aorta at 8 + 4 w [22]. By using the same analysis strategy as above, we obtain the differential metabolites in ApoE^−/−^ mice at 8 + 4 w (Figure 3B,E). The metabolites mentioned above, that had obvious changes at 8 w, still maintained their changes except acetoacetate (Table 1). Additionally, compared with C57 group, the metabolite of citrate decreased at 8 + 4 w. It was found that the stage of 8 + 8 w (severe atherosclerosis stage) shared the same metabolic characteristics as 8 + 4 w (Table 1), which indicated the metabolic markers related to AS.

### 3.4. Metabolic Regulation of SAP Deficiency in ApoE^−/−^ Mice

In order to clarify the metabolic regulation of SAP deficiency on AS, OPLS-DA was performed on ApoE^−/−^ mice and ApoE^−/−^;SAP^−/−^ mice. Figure 4A showed the obvious grouping between these two groups at 8 w, which demonstrated the significant metabolic changes due to SAP deficiency. From Figure 4D, we can see that there were 10 metabolites with VIP > 1. Based on the statistical analysis of these 10 metabolites, it was found that 6 metabolites had significant changes in ApoE^−/−^;SAP^−/−^ mice compared with ApoE^−/−^ mice (Table 1). Furthermore, the five metabolites acetoacetate, acetate, pyruvate, choline and VLDL + LDL, were regulated back to normal levels in C57 mice at 8 w. It is worth noting that tyrosine was also up-regulated at 8 w by SAP deficiency, but it had no changes at 8 + 4 w and 8 + 8 w.

Similar metabolic differences between ApoE^−/−^ mice and ApoE^−/−^;SAP^−/−^ mice also existed in 8 + 4 and 8 + 8 w. Combined with VIP values and statistical analysis, it was found that SAP deficiency continuously regulated the serum acetate, pyruvate, choline and VLDL + LDL, which were regulated back to normal levels. Therefore, the four metabolites of acetate, pyruvate, choline and VLDL + LDL were denoted as the metabolic markers of SAP deficiency.

The OPLS-DA analysis results in Figure 5 showed that the serum metabolism of ApoE^−/−^;SAP^−/−^ mice and C57 mice were still different in the three periods. In combination with the VIP score map and statistical analysis, we found that the expression of formate, tyrosine, fumarate, histidine and citrate in the serum of ApoE^−/−^;SAP^−/−^ mice was similar to that in ApoE^−/−^ mice, but significantly different from that in C57 mice. Therefore, we concluded that SAP deficiency has little effect on the regulation of these metabolites in the progression of atherosclerosis.

**Figure 6:**
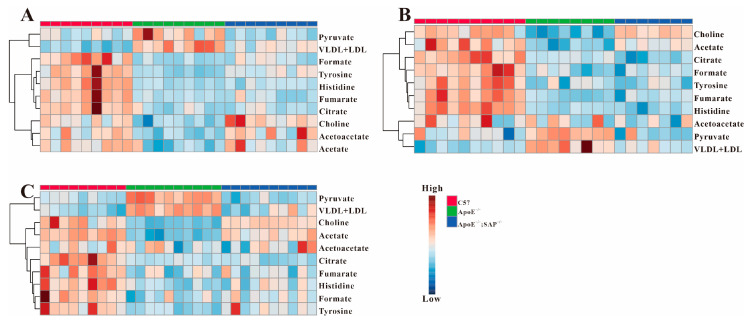
Heatmaps of all metabolic features in three groups. (**A**) metabolic profiles at 8 w; (**B**) metabolic profiles at 8 + 4 w and (**C**) metabolic profiles at 8 + 8 w.

Figure 6 shows the heatmap of metabolites in Table 1, in which the metabolic changes can be intuitively seen. From the cluster analysis, the changes of the four metabolites caused by SAP deficiency can be clearly observed, where VLDL + LDL and pyruvate showed a decrease, while acetate and choline increased significantly in the ApoE^−/−^;SAP^−/−^ group.

**Figure 7:**
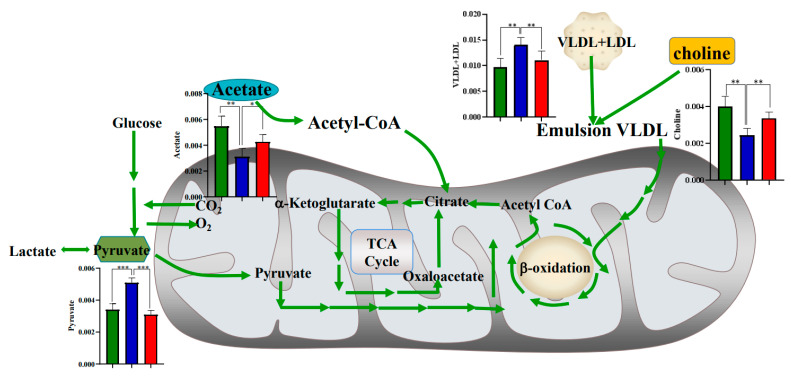
Metabolic regulatory network induced by SAP deficiency in ApoE^−/−^ model. The histogram results of statistical analysis were from the data in 8 + 8 w. ■ C57; ■ ApoE^−/−^; ■ ApoE^−/−^;SAP^−/−^. * *p* < 0.05, ** *p* < 0.01, *** *p* < 0.001.

The regulated metabolic pathways induced by SAP deficiency were given in Figure 7, where it was revealed that pyruvate, acetate, choline and VLDL + LDL were regulated during the whole progression of AS, especially at the period without obvious atherosclerosis (8 w). This result demonstrated that the metabolic regulation of SAP deficiency is one of the important mechanisms for its inhibition of atherosclerosis, and this metabolic regulation occurred before the appearance of obvious atherosclerotic plaque.

### 3.5. High Fat Diet Has No Effect on the Metabolic Regulation of SAP Deficiency

**Figure 8:**
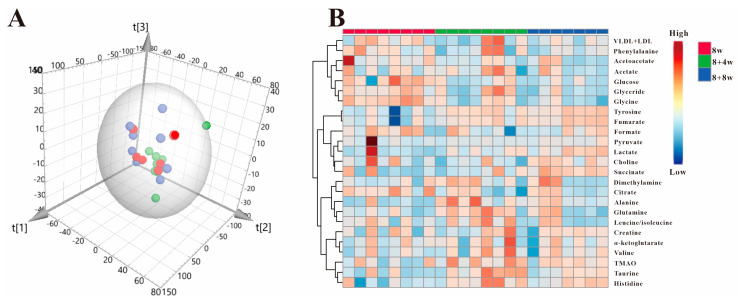
(**A**) PCA scores plots for ^1^H-NMR spectra data of SAP^−/−^ mice serum. ● 8 w; ● 8 + 4 w; ● 8 + 8 w; R^2^X = 0.975, Q^2^ = 0.816. (**B**) Heatmap of SAP^−/−^ mice in three periods.

As mentioned above, all mice were fed with a high-fat diet throughout the experimental cycle. In order to analyze whether the metabolic changes regulated by SAP deletion were related to a high-fat diet, we conducted PCA on the data from SAP^−/−^ mice at 8 w, 8 + 4 w and 8 + 8 w. The results are shown in Figure 8A. The 3D scatter plot of PCA could not distinguish the mice in these three stages, which indicated that the overall metabolism of SAP^−/−^ mice in these three stages was similar, and there were no significant metabolic differences. Figure 8B showed the results of hierarchical cluster analysis for SAP^−/−^ mice which showed that there were no obvious metabolic changes in the three stages. All the results revealed that the metabolic changes caused by SAP deficiency have nothing to do with high-fat diet, which is caused by gene deletion.

## 4. Discussion

ApoE^−/−^ is considered to be a good model for the study of atherosclerosis, the pathological process of which is similar to that of humans [23]. Atherosclerosis is characterized by disorders of glucose and lipid metabolism. In our previous study, it was proved that SAP knockout can inhibit the progression of atherosclerosis [8], but its metabolic regulation on AS was unknown. In this report, we clarified the metabolic regulation mechanism of SAP knockout in ApoE^−/−^;SAP^−/−^ mice by use of ^1^H−NMR-based metabonomics, which explained the role of SAP deficiency on AS from the perspective of metabolism.

It was revealed that pyruvate, acetate, choline and VLDL + LDL were regulated during the whole progression of AS, especially at the period without obvious atherosclerotic plaque formation (8 w). This result demonstrated that the metabolic regulation of SAP deficiency is one of the important mechanisms for its inhibition of atherosclerosis, and the metabolic regulation is prior to the appearance of obvious atherosclerotic plaques.

Studies have shown that the pathological process of atherosclerosis is closely related to the severity of plasma hyperlipidemia. Cholesterol exists in several forms including HDL, and VLDL + LDL, and the plasma concentration of VLDL + LDL is clinically used as one of the criteria for the diagnosis of hypercholesterolemia [24]. It was found that VLDL + LDL in ApoE^−/−^ mice increased significantly with the increase of the high-fat diet cycle compared with C57 mice, while in ApoE^−/−^;SAP^−/−^ mice, the levels of VLDL + LDL were not statistically different from those in C57 mice, which indicated that SAP deficiency could improve the atherosclerosis by regulating the metabolism of VLDL + LDL. Choline has good emulsifying properties, which can prevent the deposition of cholesterol in the inner wall of blood vessels and remove some of the deposits, and improve the absorption and utilization of fat [25]. So, it has the effect of preventing cardiovascular disease [26]. Some studies believed that deletion of the SAP gene could improve atherosclerosis through its effect of promoting cholesterol efflux [6]. Our study found that with the increase in high-fat diet cycles, the level of serum choline in ApoE^−/−^ mice decreased significantly compared with C57 mice. However, the choline in ApoE^−/−^;SAP^−/−^ mice is not statistically different from that in C57 mice. Therefore, we concluded that SAP deficiency may directly regulate the metabolism of VLDL + LDL, and it can also promote the metabolism of cholesterol by increasing the content of choline in serum.

Acetate can be metabolized to coenzyme acetate (CoA) and enters the TCA cycle [27], and it has been used to assess TCA cycle activity in the myocardium [28]. Our study found that the serum acetate content in ApoE^−/−^ mice was significantly lower than that in C57 mice, while the serum acetic acid content in ApoE^−/−^;SAP^−/−^ mice was similar to that in C57 mice at the three periods. There was no statistical difference between ApoE^−/−^;SAP^−/−^ and C57 mice. However, we found that some important intermediates of the TCA cycle were not regulated by SAP deficiency, such as fumarate, citrate, succinate and α-ketoglutarate. The TCA cycle not only participates in the aerobic oxidation of glucose, but also is central to the regulation of energy homeostasis and cellular metabolism [29]. The tricarboxylic acid cycle is the final metabolic pathway of the three major nutrients (glucose, lipid and amino acid), and also the hub for the metabolic connection of the three major nutrients [30]. Fumarate, succinate, citrate and α-ketoglutarate are important intermediates in the TCA cycle and play crucial roles in cellular aerobic respiration [31]. Shiomi et al. demonstrated that the serum citrate level is closely related to the pathological process of atherosclerosis [32]. In a rabbit model of atherosclerosis, Peng, J.B. et al. [33] found that citrate levels continued to decrease throughout the progress of atherosclerosis. Our results also proved this point of view. The relative concentrations of fumarate and citrate in the serum of ApoE^−/−^ and ApoE^−/−^;SAP^−/−^ mice were always lower than those in C57 mice. It has been shown that in the inflammatory environment of atherosclerosis, more glycolytic pathways are activated and TCA circulation is impaired [34]. Restriction of the TCA cycle flux also further affects cell metabolic checkpoints such as phosphorylation and stability of AMP-activated protein kinase (AMPK), increasing the risk of atherosclerotic disease [35]. Therefore, we believe that although SAP deficiency can improve the disorder of acetate in ApoE^−/−^ mice, it cannot change the disorder of TCA circulation in atherosclerosis only by regulating acetate, which may be related to the negative feedback regulation of the body itself.

Pyruvate can realize the mutual conversion of sugars, fats and amino acids in the body through the acetyl CoA and tricarboxylic acid cycles [36]. It is an important intermediate product in the metabolism and mutual conversion of various substances in biological cells [37]. The network of 20 metabolic biomarkers constructed by Tian et al. [38] shows that pyruvate metabolism is seriously disturbed in the process of atherosclerosis. We found that serum pyruvate in ApoE^−/−^ mice was significantly higher than that in C57 group, but there was no statistical difference between ApoE^−/−^;SAP^−/−^ and C57 mice, indicating that the SAP knockout may improve the metabolic disorder of pyruvate in the process of atherosclerosis to some extent.

There are significant metabolic differences between ApoE^−/−^ mice and C57 mice. Deletion of the SAP gene can significantly change the expression of some differential metabolites, especially pyruvate, acetate, choline and VLDL + LDL, but there are still some differential metabolites that have not been affected. Our study found that the four metabolites of formate, tyrosine, fumarate and histidine in ApoE^−/−^ mice showed a downward trend in three periods, but SAP deficiency could not bring them back to the expression levels in C57 mice. The same is true for the metabolite of citrate. Although it had significant changes in ApoE^−/−^ at 8 + 4 w and 8 + 8 w, SAP deficiency had no obvious regulatory effect on citrate. Importantly, it should not be overlooked whether the deletion of the SAP gene causes the expression of proteins associated with the progression of AS.

In this study, we used an ^1^H-NMR-based metabonomics approach to explore the metabolic regulation mechanism of SAP knockout in ApoE^−/−^;SAP^−/−^ mice. Despite its unsatisfactory sensitivity, NMR spectra still plays a key role in metabonomics research because of its high repeatability, non-destructiveness and non-invasiveness, which makes our research data reliable. On the whole, our study elucidated the metabolic regulation of atherosclerosis by SAP knockout from the timeline. However, NMR method also has limitations, such as low sensitivity and the small number of metabolites that can be characterized. Later, we will conduct a more comprehensive study on the metabolic regulation of SAP in combination with MS technology.

## 5. Conclusions

In conclusion, our report has shown the metabolic regulation effects of SAP efficiency on atherosclerosis in ApoE^−/−^ mice. It was demonstrated that the metabolites of pyruvate, acetate, choline and VLDL + LDL were regulated during the whole progression of AS, especially at the period without obvious atherosclerotic plaques, indicating that SAP’s regulation of metabolism started before the appearance of atherosclerosis, and this metabolic regulation has nothing to do with a high-fat diet.

## Figures and Tables

**Table 1:** Statistical analysis of potential biochemical markers in serum of C57 mice, ApoE^−/−^ mice and ApoE^−/−^;SAP^−/−^ mice.

Metabolites	δ ^1^H (ppm)	Pathway	8 w			8 + 4 w			8 + 8 w		
			ApoE^−/−^ vs. C57	ApoE^−/−^;SAP^−/−^ vs. ApoE^−/−^	ApoE^−/−^;SAP^−/−^ vs. C57	ApoE^−/−^ vs. C57	ApoE^−/−^;SAP^−/−^ vs. ApoE^−/−^	ApoE^−/−^;SAP^−/−^ vs. C57	ApoE^−/−^ vs. C57	ApoE^−/−^;SAP^−/−^ vs. ApoE^−/−^	ApoE^−/−^;SAP^−/−^ vs. C57
Formate	8.46	Ketone metabolism	↓ **	-	↓ *	↓ *	-	↓ *	↓ **	-	↓ *
Tyrosine	6.89, 7.81	Amino acid metabolism	↓ ***	↑ **	↓ *	↓ ***	-	↓ **	↓ **	-	↓ *
Fumarate	6.51	Energy metabolism	↓ ***	-	↓ **	↓ ***	-	↓ **	↓ **	-	↓ **
Histidine	7.04, 7.74	Amino acid metabolism	↓ ***	-	↓ **	↓ ***	-	↓ **	↓ ***	-	↓ **
Citrate	2.54	Energy metabolism	-	-	-	↓ **	-	↓ *	↓ *	-	↓ **
Acetoacetate	2.28	Energy metabolism	↓ **	↑ **	-	-	-	-	-	-	-
Acetate	1.92	Ketone metabolism	↓ *	↑ *	-	↓ **	↑ **	-	↓ **	↑ *	-
Pyruvate	2.37	Energy metabolism	↑ **	↓ **	-	↑ *	↓ **	-	↑ ***	↓ ***	-
Choline	3.2	Lipid metabolism	↓ **	↑ *	-	↓ **	↑ *	-	↓ **	↑ **	-
VLDL + LDL	0.88, 1.28	Lipid metabolism	↑ **	↓ **	-	↑ **	↓ *	-	↑ **	↓ **	-

Note: ↑, the increase; ↓, the decrease; * *p* < 0.05, ** *p* < 0.01, *** *p* < 0.001, -, no statistical change.

## Data Availability

Data are contained within the article or Supplementary Materials.

## Supplementary Materials

The following supporting information can be downloaded at: https://www.mdpi.com/article/10.3390/metabo12121278/s1, Figure S1: Design illustration of the entire study, Figure S2: Model validation, substitution testing.
