# Supplementary figures and images for: 1H-NMR-Based Metabonomics Study to Reveal the Progressive Metabolism Regulation of SAP Deficiency on ApoE−/− Mice
Source: Metabolites. 2022 Dec 16;12(12):1278. doi: 10.3390/metabo12121278 (PMC9785365; doi:10.3390/metabo12121278)

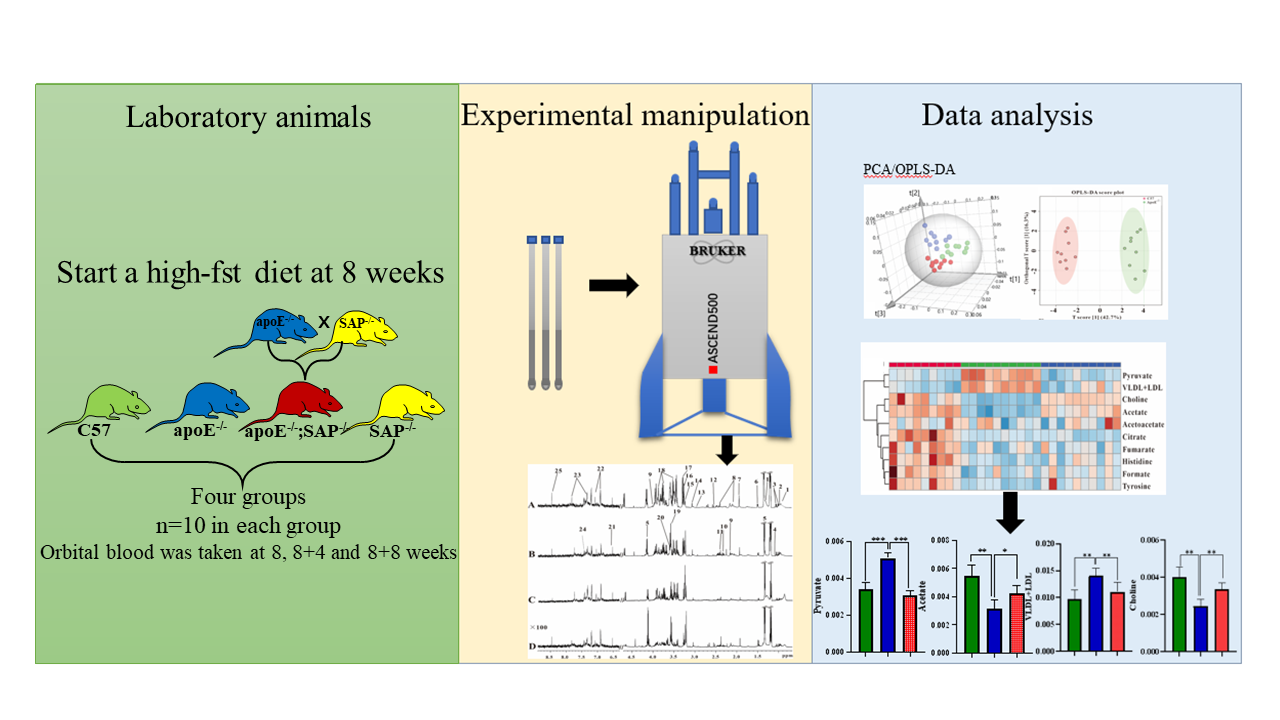

Supplement: Supplementary file 1 [file metabolites-12-01278-s001.zip › Figure S1. Design illustration of the entire study.png]

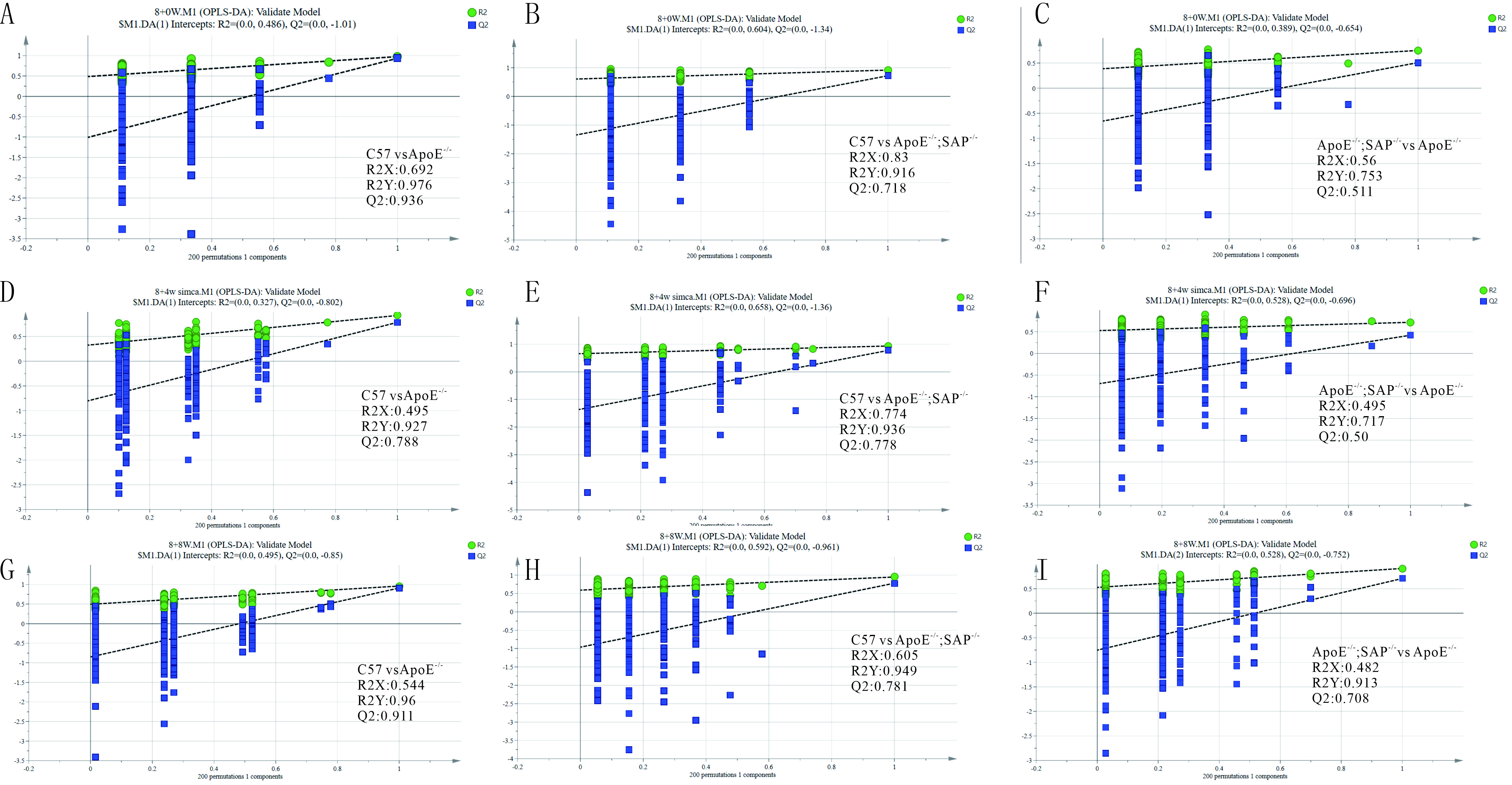

Supplement: Supplementary file 1 [file metabolites-12-01278-s001.zip › Figure S2. Model validation, substitution testing.jpg]
